# Combined Micronutrient and Microbial Inoculant Application Improves Bur Clover Yield and Quality While Reshaping Rhizosphere Microbial Communities Under Greenhouse Conditions

**DOI:** 10.3390/microorganisms14051010

**Published:** 2026-04-30

**Authors:** Guiliang Wang, Yao Liu, Chen Zhao, Haitao Zhao, Xiaoqing Qian, Juanjuan Wang

**Affiliations:** Key Laboratory of Arable Land Quality Monitoring and Evaluation (Yangzhou University), Ministry of Agriculture and Rural Affairs, Yangzhou 225127, China; wgl0520@126.com (G.W.); 13624169983@163.com (Y.L.); zhaochen1117yz@163.com (C.Z.); htzhao@yzu.edu.cn (H.Z.); qianxq@yzu.edu.cn (X.Q.)

**Keywords:** bur clover, micronutrient, compound microbial inoculant, microbial community assembly, yield, quality

## Abstract

Micronutrient limitation and rhizosphere imbalance often constrain the productivity and quality of leafy vegetables in intensively managed greenhouse soils. This study evaluated the effects of conventional fertilization (CK), micronutrient supplementation (Mi), and micronutrient supplementation combined with a compound microbial inoculant (MM) on bur clover (*Medicago polymorpha* L.) yield, quality, rhizosphere chemical properties, and soil microbial communities. Compared with CK, Mi increased yield by 26.53%, whereas MM increased yield by 40.77%. MM also significantly increased SPAD, soluble protein, and soluble sugar, while reducing plant nitrate content by 22.86%; Mi had no significant effect on nitrate reduction. MM decreased soil pH from 8.62 to 8.34 and increased EC, available P and K, water-soluble Ca, Mg, and K, and available Zn and B, indicating improved rhizosphere chemical conditions. Mantel analysis showed that yield and plant nitrate were significantly associated with several soil variables. MM also markedly reshaped rhizosphere microbial communities, with clear treatment separation for both bacteria and fungi. The bacterial community was significantly explained by selected soil variables, whereas the fungal model was not significant. Overall, micronutrient supplementation mainly promoted yield, while its combination with microbial inoculation further improved rhizosphere conditions, crop quality, and nitrate control.

## 1. Introduction

Greenhouse production systems play an important role in modern vegetable production because they enable intensive, year-round cultivation and provide high and stable economic returns [1,2]. However, long-term intensive cultivation and repeated fertilizer inputs under greenhouse conditions often lead to soil-related constraints, including nutrient imbalance, secondary salinization, and unstable product quality [3,4,5]. These changes not only limit crop productivity, but also increase the risk of excessive nitrate accumulation in edible tissues, which is a major concern for the safety and marketability of leafy vegetables [6]. Therefore, achieving high yield and improved quality while minimizing nitrate accumulation has become a key challenge for the sustainable production of leafy vegetables in greenhouse systems.

Micronutrient management is an important strategy for alleviating soil constraints and improving crop performance. Elements such as Zn, B, Mn, Fe, and Mo play essential roles in photosynthesis, enzyme activation, nitrogen metabolism, and the synthesis of quality-related compounds [7,8]. In greenhouse soils, however, the availability of these micronutrients is often limited, particularly under long-term intensive cultivation, which is frequently accompanied by soil constraints that alter rhizosphere pH and ionic balance, reduce nutrient solubility and mobility, and intensify antagonistic interactions among elements, thereby restricting plant uptake and utilization of micronutrients [7,8,9]. Accordingly, appropriate micronutrient supplementation can help improve nutrient availability and promote plant growth [10,11]. Nevertheless, the effectiveness of micronutrient application alone is often constrained when rhizosphere conditions and nutrient transformation processes remain suboptimal. For example, under neutral-to-alkaline soil conditions, the bioavailability of Zn and Fe remained limited even after micronutrient application, whereas formulations functionalized with plant growth-promoting rhizobacteria were more effective in enhancing seedling growth [12]. These findings suggest that, beyond simple nutrient input, regulation of rhizosphere processes may be necessary to achieve coordinated improvements in yield, quality, and nitrate reduction.

The rhizosphere microbiome plays a key role in nutrient mobilization, root nutrient acquisition, and plant physiological performance [13,14]. Beneficial microbial inoculants, especially consortia containing *Bacillus*, *Pseudomonas*, and *Trichoderma*, can enhance nutrient cycling and rhizosphere conditions, thereby improving nutrient use efficiency, reshaping rhizosphere microbial assembly, and ultimately promoting plant performance [15,16,17]. However, existing studies have mainly focused on yield or individual quality traits, whereas the relationships among rhizosphere nutrient status, microbial community assembly, and crop performance remain poorly understood [18,19,20]. In particular, it is still unclear whether adding a microbial consortium to micronutrient supplementation can further improve crop quality and reduce nitrate accumulation through changes in rhizosphere nutrient availability and microbial community structure.

Bur clover (*Medicago polymorpha* L.) is an annual legume valued for its rapid growth, high biomass, nutritional value, and good palatability, and is widely used as a vegetable, forage, and cover crop [21]. Its strong adaptability, biological nitrogen fixation, and soil-improving capacity further support its role in sustainable production systems [22]. Accordingly, bur clover is widely cultivated in eastern China as a fresh leafy vegetable with high market value and desirable eating quality [23]. This study was conducted in a greenhouse bur clover production system with three treatments: conventional fertilization (CK), micronutrient supplementation (Mi), and micronutrient supplementation combined with a compound microbial inoculant (MM). The aim of this study was to evaluate the effects of different amendment strategies on crop yield, quality, and soil quality, and to identify the optimal management practice.

## 2. Materials and Methods

### 2.1. Experimental Site

The greenhouse soil experiment was conducted from 2023 to 2024 at the Yangcao Planting Cooperative in Yangzhong City, Jiangsu Province, China (32°09′51″ N, 119°52′12″ E). The site is characterized by a subtropical monsoon climate, with a mean annual temperature of 15.4 °C, annual precipitation of 1057 mm, annual sunshine duration of 2135 h, and a frost-free period of 227 d. The experimental soil was of loamy clay texture. Before treatment application, soil samples were collected from three greenhouses using an S-shaped sampling pattern along the length of each greenhouse. Five sampling points were taken from the 0–20 cm soil layer in each greenhouse with a soil auger, resulting in a total of 15 sampling points. These 15 soil samples were thoroughly mixed to form one composite sample for the determination of the initial soil physicochemical properties. The initial physicochemical properties of the experimental soil before treatment application are presented in the Pre-treatment column of Table 1.

### 2.2. Experimental Design

Three greenhouse tunnels with uniform previous-crop growth were selected for the experiment. Each tunnel measured 4 m × 65 m and was equally divided into three plots, resulting in a total of nine plots. The experiment consisted of three treatments with three replicates and was arranged in a single-factor randomized block design. The treatments were as follows: (1) CK, conventional production practice, with 300 kg ha^−1^ organic fertilizer, 30 kg N ha^−1^, 90 kg P_2_O_5_ ha^−1^, and 150 kg K_2_O ha^−1^; (2) Mi, based on CK, supplemented with micronutrients including 225 kg MgSO_4_ ha^−1^, 30 kg ZnSO_4_ ha^−1^, 15 kg borax ha^−1^, 45 kg MnSO_4_ ha^−1^, 60 kg FeSO_4_ ha^−1^, and 2.25 kg ammonium molybdate ha^−1^; (3) MM, based on Mi, further supplemented with 900 kg ha^−1^ microbial agent containing *Bacillus amyloliquefaciens*, *Bacillus subtilis*, *Bacillus licheniformis*, *Pseudomonas fluorescens*, and *Trichoderma harzianum*, which was obtained from Nanjing Sinong High-Tech Co., Ltd. (Nanjing, China). Other field management practices followed those commonly adopted by local high-yield growers under greenhouse conditions.

The bur clover cultivar was ‘Wenling Daye Huanghuacaotou’, provided by Wenling Shennong Seed Co., Ltd., Seeds, Wenling, China were sown by broadcast seeding. Seeds were sown on 15 September by broadcast seeding at a rate of 180 kg ha^−1^, and the shoots were harvested multiple times from October to the following April according to marketable maturity.

### 2.3. Measurements and Analyses

#### 2.3.1. Bur Clover Yield and Quality Analysis

After sowing, all marketable bur clover shoots in each plot were harvested at approximately 15-day intervals using a grass cutter. The harvested portion included the entire edible shoot, consisting of intact leaves together with 2–8 cm of stem. The fresh weight of the harvested biomass was measured and recorded as yield.

For quality determination, representative plants with uniform growth were sampled from each plot during the yield measurements on 15 December 2023 and 30 March 2024. At the same time, SPAD values (Soil and Plant Analysis Development chlorophyll meter reading) were measured on 10 fully expanded leaves with uniform growth in each plot using a handheld chlorophyll meter (SPAD-502Plus, Konica Minolta, Tokyo, Japan). Fresh shoots were immediately placed into sealed plastic bags lined with moist gauze and transported to the laboratory. The samples were then gently wiped clean with a soft damp cloth and stored at 0–4 °C prior to analysis. Fresh samples were used to determine soluble protein, soluble sugar, and nitrate contents [24].

#### 2.3.2. Soil Sampling and Physicochemical Analyses

On 30 March 2024, rhizosphere-adjacent soil samples were collected from the 0–20 cm soil layer near bur clover roots using a soil auger. Five subsamples were collected from each plot following a five-point S-shaped sampling method and then thoroughly mixed to obtain one composite soil sample per plot. Each composite sample was divided into two portions: one portion was immediately stored at low temperature for microbial diversity analysis, and the other was transported to the laboratory, air-dried, and used for the analysis of soil physicochemical properties. Soil pH and EC were measured in 1:2.5 and 1:5 soil-to-water suspensions using a pH meter (FiveEasy F20, Mettler Toledo, Greifensee, Switzerland) and a conductivity meter (DDS-307A, INESA Scientific Instrument Co., Ltd., Shanghai, China), respectively. Soil organic matter (SOM) was determined by potassium dichromate oxidation with external heating. Nitrate-N (NO3−-N), ammonium-N (NH4+-N), available P (AP), available B (AB), and available Mo (AMo) were determined using a UV–Vis spectrophotometer (UV-2600i Plus, Shimadzu, Kyoto, Japan) by UV spectrophotometry, the indophenol blue method, the molybdenum antimony colorimetric method, the curcumin colorimetric method, and the potassium thiocyanate colorimetric method, respectively. Available K (AK) was determined by flame photometry (FP6410, INESA Scientific Instrument Co., Ltd., Shanghai, China). Water-soluble Ca (WSCa) and Mg (WSMg), as well as DTPA-extractable Fe (AFe), Mn (AMn), Cu (ACu), and Zn (AZn), were determined using an atomic absorption spectrometer (PinAAcle 900T, PerkinElmer, Waltham, MA, USA). All soil analyses were performed according to standard procedures described by Bao (2000) [25].

#### 2.3.3. Soil Microbial Community Analysis

Total genomic DNA was extracted from 0.5 g of fresh soil using the PowerSoil DNA Isolation Kit (MoBio, QIAGEN, Carlsbad, CA, USA) according to the manufacturer’s instructions. The bacterial 16S rRNA gene was amplified with primers 338F/806R, and the fungal ITS region was amplified with primers ITS1/ITS2. PCR products were verified by 2% agarose gel electrophoresis, purified using the AxyPrep DNA Gel Extraction Kit (Axygen, Union City, CA, USA), quantified with the QuantiFluor™-ST system (Promega, Madison, WI, USA), and sequenced on the Illumina MiSeq platform by Majorbio Bio-Pharm Technology Co., Ltd. (Shanghai, China).

Sequence processing and bioinformatic analyses were conducted on the Majorbio I-Sanger Cloud Platform. Operational taxonomic units (OTUs) were clustered at 97% sequence similarity, and taxonomic annotation was performed against the Silva database for bacteria and the UNITE 8.0 database for fungi. Based on the OTU table, alpha diversity indices (Sobs, Chao1, Ace, Shannon, and Simpson) were calculated, and microbial community composition was characterized at the phylum and genus levels. Principal coordinates analysis (PCoA) was used to evaluate differences in microbial community structure among treatments. Differential bacterial and fungal taxa among treatments were then identified using LEfSe (linear discriminant analysis effect size), and taxa with LDA scores > 2.0 were retained as discriminative features for further analysis. To identify the environmental factors associated with treatment-related variation in microbial community composition, redundancy analysis (RDA) was performed after variance inflation factor (VIF) screening of environmental variables; variables with VIF values < 10 were retained in the final model. Correlation heatmaps were further constructed in R (version 4.5.2; R Foundation for Statistical Computing, Vienna, Austria) to assess the associations of treatment-responsive taxa with environmental factors and plant performance traits.

### 2.4. Statistical Analysis

All original data were compiled in Microsoft Excel 2021. Statistical analyses were carried out using IBM SPSS Statistics 19.0, and figures were created with R (version 4.5.2; R Foundation for Statistical Computing, Vienna, Austria). Treatment effects were analyzed by one-way analysis of variance (ANOVA), followed by the least significant difference (LSD) test for multiple comparisons at *p* < 0.05.

## 3. Results

### 3.1. Effects of Different Treatments on Yield and Quality of Bur Clover

Compared with CK (Table 2), the Mi treatment significantly increased bur clover yield by 26.53%, while the MM treatment increased yield by 40.77%. For quality traits, the SPAD value under MM reached 44.23, which was significantly higher than those under both CK and Mi. Both Mi and MM significantly increased soluble protein content relative to CK, with no significant difference between the two treatments (mean value: 22.58 mg kg^−1^). Soluble sugar content increased from CK to Mi and then to MM. In contrast, nitrate content did not differ significantly between CK and Mi, whereas MM significantly reduced nitrate content by 22.86% compared with CK.

### 3.2. Effects of Different Treatments on Soil Basic Chemical Properties and Micronutrient Contents

Soil chemical properties and micronutrient contents differed significantly among treatments (Table 1). Soil pH decreased from 8.62 in CK to 8.52 in Mi and 8.34 in MM, whereas EC increased from 222.33 to 261.00 and 385.33 mS·cm^−1^, respectively. Soil organic matter remained unchanged among treatments (36.64–37.19 g·kg^−1^). Compared with CK (77.92 mg·kg^−1^), nitrate N was increased by Mi and MM to 97.08 and 93.33 mg·kg^−1^, respectively. Ammonium N showed no difference between CK and Mi, but declined to 0.72 mg·kg^−1^ under MM. MM exhibited the maximum contents of available P and available K (51.72 and 99.85 mg·kg^−1^), which were higher than those in CK (25.99 and 60.94 mg·kg^−1^). Water-soluble Ca and Mg showed a similar increase, from 197.49 and 44.00 mg·kg^−1^ in CK to 278.96 and 76.22 mg·kg^−1^ in MM. Water-soluble K was also highest in MM (18.64 mg·kg^−1^), but lower in Mi than in CK.

Micronutrient responses differed among elements. MM decreased available Fe and Mn, while available Cu was unchanged across treatments. By contrast, MM significantly increased available Zn and B to 6.22 and 1.50 mg·kg^−1^, respectively, but Mi exhibited values similar to or lower than CK. Available Mo was reduced in Mi but showed no significant difference between CK and MM.

**Figure 1:**
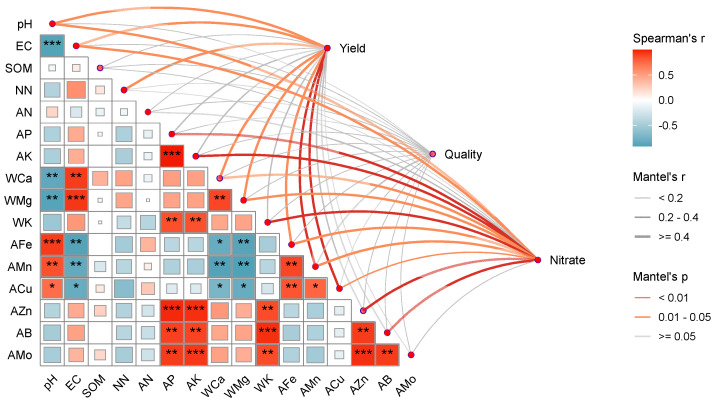
Spearman correlation matrix of soil chemical properties and Mantel test network showing the relationships of soil variables with bur clover yield, quality traits (SPAD, soluble protein, and soluble sugar), and nitrate content. Square color indicates Spearman’s correlation coefficient. Red and blue indicate positive and negative correlations, respectively. Line width represents Mantel’s r, and line color indicates Mantel’s *p* value. Asterisks indicate the significance levels of Spearman’s correlations: * *p* < 0.05, ** *p* < 0.01, and *** *p* < 0.001. Abbreviations: pH, soil pH; EC, electrical conductivity; SOM, soil organic matter; NN, nitrate nitrogen; AN, ammonium nitrogen; AP, available phosphorus; AK, available potassium; WCa, water-soluble calcium; WMg, water-soluble magnesium; WK, water-soluble potassium; AFe, available iron; AMn, available manganese; ACu, available copper; AZn, available zinc; AB, available boron; AMo, available molybdenum. Yield represents bur clover yield; Quality traits include SPAD, soluble protein, and soluble sugar; Nitrate represents nitrate content of bur clover.

Spearman correlation analysis and Mantel tests were used to assess the associations between soil properties and bur clover performance (Figure 1). AP, AK, WCa, WMg, WK, AZn, AB, and AMo were mainly positively correlated with one another, whereas pH was generally negatively correlated with EC and several nutrient-related indices, but was significantly positively correlated with AFe, AMn, and ACu. Mantel test results showed that yield and nitrate content were significantly associated with several soil variables, whereas no significant association was detected between soil properties and quality traits.

### 3.3. Effects of Different Treatments on Soil Microbial Community Characteristics

Soil microbial alpha diversity responded differently in bacterial and fungal communities (Table 3). In bacteria, MM significantly reduced richness, with Sobs, Chao1, and ACE decreasing from 4829.33, 5974.41, and 6086.02 in CK to 3624.00, 4465.96, and 4524.29, respectively. Bacterial Shannon diversity was also significantly lower under MM (6.764) than under CK (7.176), whereas the Simpson index did not differ among treatments. By contrast, Mi did not significantly affect bacterial alpha diversity.

In fungi, MM likewise significantly reduced richness, with Sobs, Chao1, and ACE declining from 340.00, 349.62, and 347.78 in CK to 147.33, 152.89, and 152.20, respectively, while Shannon and Simpson indices remained unchanged. Mi also had no significant effect on fungal alpha diversity compared with CK.

**Figure 2:**
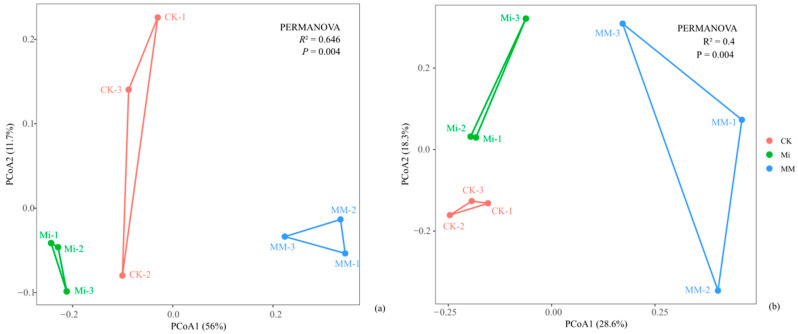
Principal coordinates analysis (PCoA) of bacterial (**a**) and fungal (**b**) communities under different treatments.

PCoA revealed clear differences in bacterial and fungal community structures among treatments (Figure 2). For bacteria, the first two axes explained 67.7% of the total variation, and the three treatments were clearly separated (*R*^2^ = 0.646, *p* = 0.004; Figure 2a). For fungi, the first two axes explained 46.9% of the total variation, and the three treatments were also clearly separated (*R*^2^ = 0.400, *p* = 0.004; Figure 2b).

**Figure 3:**
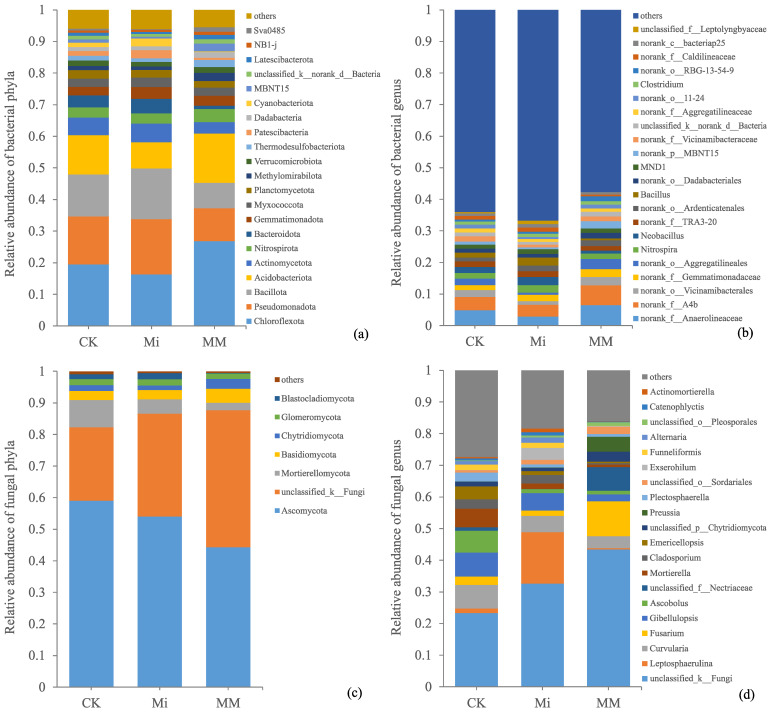
Relative abundances of bacterial phyla (**a**), bacterial genera (**b**), fungal phyla (**c**), and fungal genera (**d**) under different treatments. Taxa with a relative abundance > 1% in at least one treatment were retained, and taxa were ordered by their mean relative abundance across treatments.

At the bacterial phylum level, *Chloroflexota*, *Pseudomonadota*, *Bacillota*, and *Acidobacteriota* were dominant, together accounting for 59.81% of the total relative abundance on average (Figure 3a). Community composition varied among treatments, with *Chloroflexota* and *Acidobacteriota* relatively enriched in MM, whereas *Pseudomonadota* and *Bacillota* were more abundant in Mi. At the genus level, the dominant taxa, including *norank_f__Anaerolineaceae*, *norank_f__A4b*, *norank_o__Vicinamibacterales*, *norank_f__Gemmatimonadaceae*, and *norank_o__Aggregatilineales*, together accounted for 14.70% of the total relative abundance and generally showed higher abundances in MM than in CK (Figure 3b).

For fungi, *Ascomycota*, *unclassified_k__Fungi*, and *Mortierellomycota* were the dominant phyla, contributing 75.39% of the total relative abundance on average (Figure 3c). Compared with CK, MM increased *unclassified_k__Fungi* but decreased *Ascomycota* and *Mortierellomycota*. At the genus level, *unclassified_k__Fungi*, *Leptosphaerulina*, *Curvularia*, *Fusarium*, *Gibellulopsis*, and *Ascobolus* were the major taxa, together accounting for 55.98% of the total relative abundance (Figure 3d). Among them, *Fusarium* and *unclassified_f__Nectriaceae* were enriched in MM, whereas *Leptosphaerulina* was most abundant in Mi.

Based on the differences in community composition shown in Figure 3, LEfSe analysis was further used to identify which microbial taxa differed significantly among treatments.

**Figure 4:**
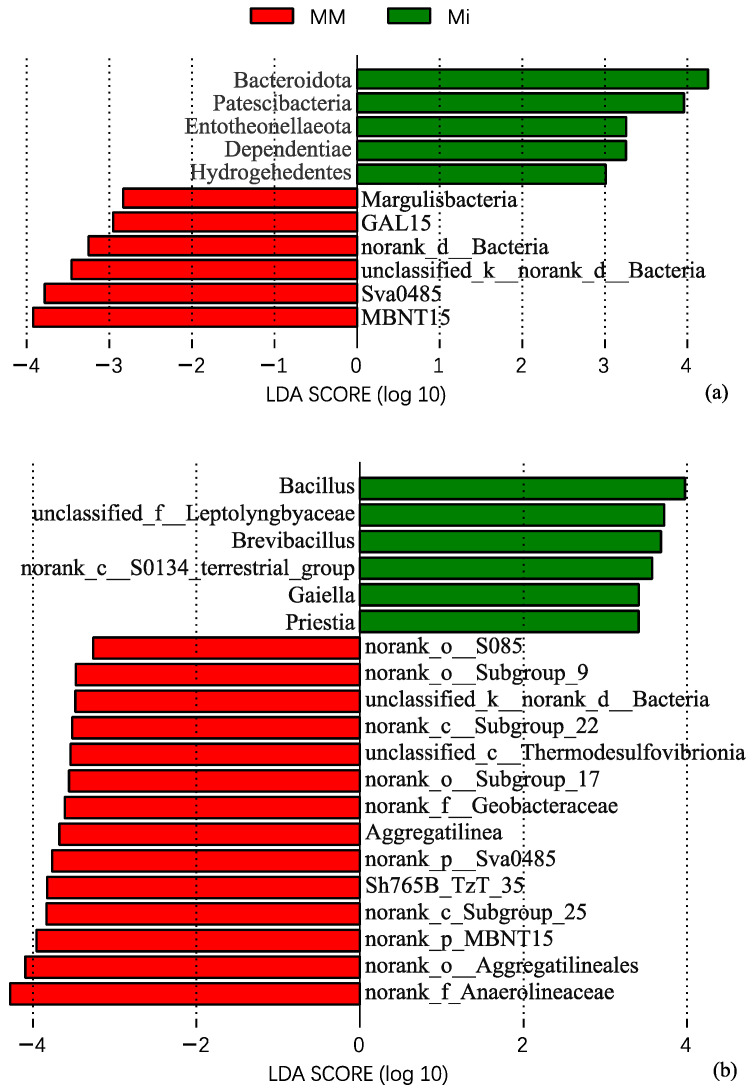
Linear discriminant analysis (LDA) scores of differentially abundant bacterial phyla (**a**) and genera (**b**) identified by LEfSe among treatments. Only taxa with LDA scores >2 are shown, and the top 20 genera are shown in panel (**b**).

LEfSe analysis identified distinct bacterial biomarkers among treatments, whereas no taxon with an LDA score > 2 was detected in CK (Figure 4). At the phylum level (Figure 4a), Mi was characterized by *Bacteroidota*, *Patescibacteria*, *Entotheonellaeota*, *Dependentiae*, and *Hydrogenedentes*, while MM was characterized by *Margulisbacteria*, *GAL15*, *norank_d__Bacteria*, *unclassified_k__norank_d__Bacteria*, *Sva0485*, and *MBNT15*. At the genus level (Figure 4b), *Bacillus, unclassified_f__Leptolyngbyaceae*, *Brevibacillus*, *norank_c__S0134_terrestrial_group*, *Gaiella*, and *Priestia* were enriched in Mi, whereas more differential genera were enriched in MM than in Mi.

**Figure 5:**
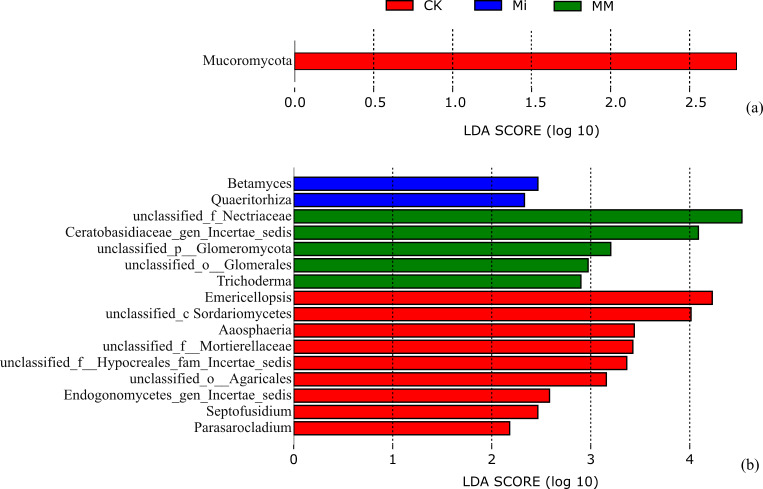
Linear discriminant analysis (LDA) scores of differentially abundant fungi phyla (**a**) and genera (**b**) identified by LEfSe among treatments. Only taxa with LDA scores >2 are shown.

LEfSe analysis identified distinct fungal biomarkers among treatments (Figure 5). At the phylum level, *Mucoromycota* was the only differential taxon with an LDA score > 2 and was associated with CK (Figure 5a). At the genus level, *Betamyces* and *Quaeritorhiza* were enriched in Mi, several taxa including *unclassified_f__Nectriaceae*, *Ceratobasidiaceae_gen_Incertae_sedis*, *unclassified_p__Glomeromycota*, *unclassified_o__Glomerales*, and *Trichoderma* were enriched in MM, whereas most differential genera were associated with CK (Figure 5b).

**Figure 6:**
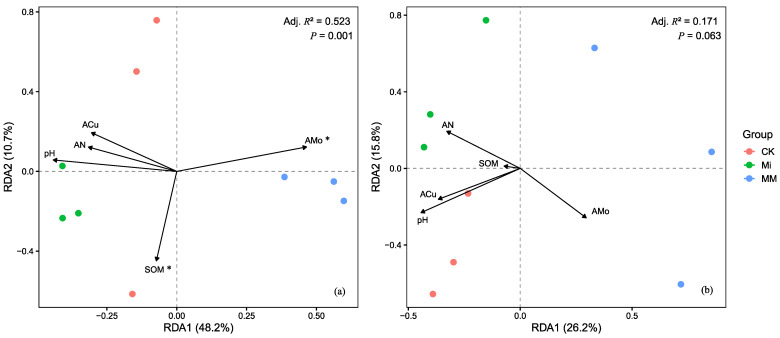
Redundancy analysis (RDA) of bacterial (**a**) and fungal (**b**) genus-level communities under different treatments based on VIF-selected environmental variables. Asterisks indicate significant explanatory environmental variables in the RDA based on permutation tests (*p* < 0.05).

Based on the observed shifts in microbial community composition, RDA was further conducted to determine which environmental factors were associated with the treatment-related differences (Figure 6). After VIF screening, pH, SOM, AN, ACu, and AMo were retained as explanatory variables in the RDA. RDA showed that the bacterial genus-level community was significantly affected by the selected environmental variables, with the first two axes explaining 58.9% of the variation (RDA1 = 48.2%, RDA2 = 10.7%) (Figure 6a). The bacterial model was significant (Adj. R^2^ = 0.523, *p* = 0.001), with MM samples distributed mainly along the positive side of RDA1 and positively associated with AMo, whereas CK and Mi samples were located mainly on the negative side of RDA1 and were more closely related to pH, AN, ACu, and SOM; among these variables, AMo and SOM were significant explanatory factors.

For fungi (Figure 6b), the first two axes explained 42.0% of the variation, but the overall model was not significant (Adj. R^2^ = 0.171, *p* = 0.063), suggesting a relatively weak relationship between fungal community composition and the selected environmental variables.

**Figure 7:**
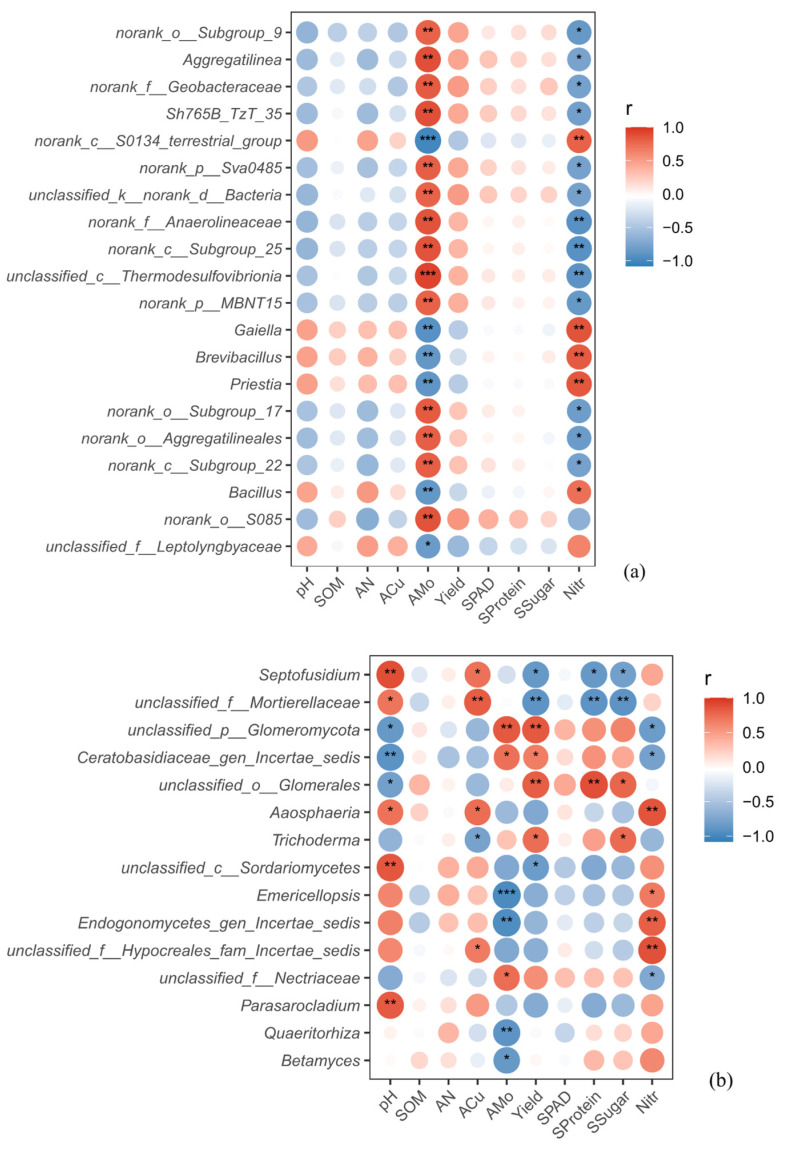
Spearman correlation heatmaps showing the associations of bacterial (**a**) and fungal (**b**) genera with LDA scores > 2 with soil environmental factors, yield, and quality traits, with only the top 20 bacterial differential genera shown. Asterisks indicate the significance levels of Spearman’s correlations: * *p* < 0.05, ** *p* < 0.01, and *** *p* < 0.001.

The treatment-responsive taxa identified by LEfSe were further used in the correlation analysis to assess their associations with environmental factors and plant performance (Figure 7). For bacteria (Figure 7a), two contrasting groups could be identified. Genera such as *norank_o__Subgroup_9*, *Aggregatilinea*, and *norank_f__Geobacteraceae* were positively correlated with AMo, yield, SPAD, soluble protein, and soluble sugar, but negatively correlated with pH, SOM, AN, ACu, and nitrate. In contrast, *Gaiella*, *Brevibacillus*, and *Bacillus* showed the opposite trend, being positively associated with pH, SOM, AN, and nitrate, while negatively correlated with AMo and the plant performance-related traits.

A similar separation was also observed for fungi (Figure 7b). Genera including *unclassified_p__Glomeromycota*, *unclassified_o__Glomerales*, and *unclassified_f__Nectriaceae* were positively associated with AMo, yield, SPAD, soluble protein, and soluble sugar, but negatively associated with pH, SOM, AN, ACu, and nitrate. By contrast, *Septofusidium*, *unclassified_f__Mortierellaceae*, and *Aaosphaeria* tended to show positive correlations with pH and ACu, but weaker or negative correlations with AMo and most plant performance variables.

## 4. Discussion

This study showed that, relative to CK, Mi increased bur clover yield by 26.53%, whereas MM increased yield by 40.77%. In addition, MM significantly increased SPAD values, soluble protein, and soluble sugar contents, while significantly reducing nitrate accumulation in plants; by contrast, Mi had no significant effect on nitrate content. These results indicate that Mi primarily promoted yield formation, whereas MM further achieved the combined benefits of higher yield and improved quality. This may be explained by the fact that MM, unlike Mi, received an additional microbial consortium; thus, its superior performance was likely driven by the additive effects of microbial inoculation and micronutrient supplementation, as beneficial microbial consortia can enhance nutrient acquisition and plant performance, particularly when combined with micronutrient nutrition inputs [26,27,28].

Compared with CK and Mi, MM significantly decreased soil pH and increased EC, AP, AK, water-soluble Ca, Mg and K, as well as available Zn and B. As shown in Figure 1, pH was generally negatively correlated with EC, AP, AK, WCa, WMg, WK, AZn, AB, and AMo, indicating that changes in soil acidity were closely linked to shifts in nutrient availability. In the present soil, the initial pH was 8.09, which increased to 8.62 under CK at harvest, whereas MM reduced it to 8.34. Although the decrease was moderate, under alkaline greenhouse soil conditions such a reduction in pH may still enhance the availability of P and certain micronutrients, thereby improving nutrient supply in the rhizosphere [8,29,30]. Figure 1 also shows that bur clover yield and nitrate content, rather than the quality traits as a whole, were significantly associated with several soil variables in the Mantel analysis. This suggests that the yield-promoting effect of MM was not attributable to the increase in a single nutrient, but rather to an overall improvement in the rhizosphere chemical environment. A moderate decrease in pH may help alleviate the constraints of alkaline soil conditions on nutrient availability, whereas the changes in EC and the concentrations of Ca, Mg, Mn, and Cu likely reflect improved supplies of plant-available nutrients and a more balanced ionic environment in the rhizosphere. Such conditions are more favorable for root nutrient uptake and assimilation, which may further promote chlorophyll formation, photosynthate accumulation, and protein synthesis, ultimately contributing to higher yield [8,31,32].

MM increased soil nitrate-N availability and soluble protein content in plants while significantly reducing nitrate accumulation in bur clover tissues. This may be because MM not only enhanced the supply of inorganic N in soil, but also promoted N uptake, translocation, and assimilation in plants, thereby favoring the conversion of inorganic N into organic N compounds such as proteins rather than its accumulation as nitrate in plant tissues. Notably, MM was also associated with higher soil Mo availability, which may have further contributed to this response, given the well-established role of Mo in nitrate reduction and N assimilation, as well as in promoting amino acid and protein synthesis while reducing nitrate accumulation in leaves [33,34]. From a production perspective, this finding is important. MM increased bur clover yield while reducing nitrate accumulation in plant tissues, indicating a greater potential to balance high productivity with the safety requirements of fresh vegetable production. Mantel analysis further showed that quality traits, including SPAD, soluble sugar, and soluble protein, were not significantly correlated with soil nutrient factors overall, suggesting that quality improvement was not driven solely by nutrient availability, but more likely resulted from the combined effects of the soil environment, plant physiological status, and rhizosphere microecology [35,36,37]. Therefore, the improvement in bur clover quality under MM was likely attributable to rhizosphere environmental optimization and coordinated carbon and nitrogen metabolism.

MM also markedly reshaped the soil microbial community, reducing the richness of both bacterial and fungal communities while causing only limited changes in evenness. This suggests that MM imposed stronger selective pressure in the rhizosphere, filtering out part of the marginal taxa while enriching groups better adapted to MM conditions or more closely associated with plant performance. In intensively managed greenhouse soils, such reduced richness but stronger directional assembly does not necessarily indicate weakened ecological function, but may instead reflect a more efficient and better-adapted rhizosphere microbiome [38,39,40]. The clear separation of bacterial and fungal communities in the PCoA further confirms that MM substantially altered community composition. Consistently, both taxonomic composition (Figure 3) and LEfSe analyses (Figure 4 and Figure 5) showed that MM enriched a wider range of differential bacterial and fungal taxa than Mi, indicating that MM was more effective than micronutrient supplementation alone in reconstructing a rhizosphere microbial community with stronger selectivity. Similar findings have been reported in previous studies, where microbial inoculation, especially when combined with fertilizer inputs or applied as microbial consortia, more strongly reshaped rhizosphere community structure and enriched beneficial taxa than fertilizer application alone [41,42].

As shown by the RDA (Figure 6), RDA showed that the selected soil variables explained a substantial proportion of the variation in the bacterial community at the genus level, whereas the fungal model was not significant, indicating that under the present conditions, bacterial communities were more sensitive than fungal communities to MM-induced changes in the rhizosphere environment. This may be because, compared with fungi, bacteria generally have faster growth and turnover rates and depend more strongly on labile nutrients, making them more sensitive to fertilization and changes in soil chemistry [43,44,45]. The close association between AMo and MM samples is particularly noteworthy, as Mo is an essential micronutrient involved in nitrogen metabolism in both plants and microorganisms, participating in nitrate reduction and nitrogen assimilation. An adequate Mo supply is therefore conducive to protein synthesis, quality improvement, and reduced nitrate accumulation [33,34]. Consistent with this pattern, the correlation heatmaps (Figure 7) further suggested that MM shifted rhizosphere microbial community composition along environmental gradients characterized by higher AMo, lower pH, and lower nitrate levels, thereby promoting microbial assemblages more closely associated with improved bur clover performance. In contrast, SOM was only weakly correlated with community variation, suggesting that microbial differentiation in this study was influenced more by dynamic, plant-available nutrients and rhizosphere chemical conditions than by the relatively stable pool of total soil organic matter [46,47].

Overall, although the sole application of micronutrients increased bur clover yield under greenhouse conditions, its effects on quality improvement and nitrate reduction were limited. In contrast, combining micronutrients with a compound microbial inoculant further improved the rhizosphere environment and promoted a microbial community composition associated with superior plant performance. Although the underlying mechanisms require further investigation, the present results indicate that the combined use of micronutrients and a compound microbial inoculant is a promising management strategy for enhancing yield and quality, reducing nitrate accumulation, and improving the rhizosphere ecological environment.

## 5. Conclusions

Compared with CK, Mi increased bur clover yield by 26.53%, whereas MM produced a greater increase of 40.77%. MM also improved plant quality, with higher SPAD, soluble protein, and soluble sugar, and reduced nitrate content by 22.86% relative to CK, while Mi showed no significant effect on nitrate reduction. MM decreased pH from 8.62 to 8.34 and increased EC, available P and K, water-soluble Ca, Mg, and K, and available Zn and B, indicating a clear improvement in rhizosphere chemical conditions. Mantel analysis showed that yield and plant nitrate were significantly associated with several soil variables, whereas quality traits were not directly related to soil nutrients alone. MM also markedly altered rhizosphere microbial communities, with significant treatment separation for bacteria (R^2^ = 0.646, *p* = 0.004) and fungi (R^2^ = 0.400, *p* = 0.004); bacterial community variation was significantly explained by selected soil variables (Adj. R^2^ = 0.523, *p* = 0.001), whereas the fungal model was not significant. Therefore, the combined application of micronutrients and a compound microbial inoculant represents a promising management strategy for improving yield, quality, and production safety of bur clover in alkaline greenhouse soils. These findings further suggest that optimizing rhizosphere chemical conditions and microbial community assembly is biologically important for enhancing crop performance under intensive greenhouse cultivation. Future work should focus on validating these effects over longer terms and under different soil conditions.

## Figures and Tables

**Table 1:** Effects of different treatments on soil basic chemical properties and micronutrient contents.

Treatments	Pre-Treatment	CK	Mi	MM	*p* Value
pH	8.09	8.62 ± 0.09 a	8.52 ± 0.06 b	8.34 ± 0.06 c	0.0083
EC (ms·cm^−1^)	201.73	222.33 ± 39.32 c	261.00 ± 34.77 b	385.33 ± 60.37 a	0.0117
Soil organic matter (g·kg^−1^)	26.73	37.19 ± 3.33 a	36.96 ± 1.83 a	36.64 ± 0.67 a	0.9548
Nitrate N (mg·kg^−1^)	144.89	77.92 ± 2.41 b	97.08 ± 6.39 a	93.33 ± 6.39 a	0.0107
Ammonium N (mg·kg^−1^)	9.52	1.11 ± 0.08 a	1.20 ± 0.21 a	0.72 ± 0.10 b	0.0132
Available P (mg·kg^−1^)	24.94	25.99 ± 6.01 c	22.87 ± 10.18 b	51.72 ± 8.79 a	0.0112
Available K (mg·kg^−1^)	91.38	60.94 ± 15.96 b	48.46 ± 9.15 c	99.85 ± 15.96 a	0.0101
Water-soluble Ca (mg·kg^−1^)	167.98	197.49 ± 30.91 c	220.46 ± 16.10 b	278.96 ± 16.43 a	0.0105
Water-soluble Mg (mg·kg^−1^)	38.19	44.00 ± 7.29 c	51.71 ± 11.98 b	76.22 ± 7.46 a	0.0120
Water-soluble K (mg·kg^−1^)	5.96	7.63 ± 0.62 b	5.48 ± 3.57 c	18.64 ± 5.44 a	0.0110
Available Fe (mg·kg^−1^)	30.86	10.86 ± 0.09 a	10.43 ± 0.70 a	9.32 ± 0.26 b	0.0122
Available Mn (mg·kg^−1^)	12.17	5.85 ± 0.35 a	4.43 ± 0.93 b	3.62 ± 0.35 c	0.0114
Available Cu (mg·kg^−1^)	12.90	4.62 ± 0.81 a	4.51 ± 0.82 a	4.46 ± 0.76 a	0.1166
Available Zn (mg·kg^−1^)	6.10	1.97 ± 1.47 b	1.56 ± 1.55 b	6.22 ± 1.47 a	0.0160
Available B (mg·kg^−1^)	0.64	0.50 ± 0.00 b	0.41 ± 0.18 b	1.50 ± 0.54 a	0.0118
Available Mo (mg·kg^−1^)	0.050	0.064 ± 0.005 a	0.048 ± 0.005 b	0.066 ± 0.005 a	0.0085

Values are means ± SD, Different letters within the same row indicate significant differences among treatments (*p* < 0.05).

**Table 2:** Effects of different treatments on yield and quality of bur clover.

Treatments	Yield (kg hm^−2^)	SPAD	Soluble Protein (mg kg^−1^ FW)	Soluble Sugar (%)	Nitrate (mg kg^−1^ FW)
CK	35,346 ± 1783 c	41.58 ± 3.03 b	19.36 ± 1.08 b	3.08 ± 0.11 c	1461.82 ± 39.14 a
Mi	44,724 ± 2991 b	41.37 ± 3.38 b	22.21 ± 1.44 a	3.75 ± 0.12 b	1496.22 ± 35.1 a
MM	49,756 ± 1555 a	44.23 ± 4.11 a	22.95 ± 0.6 a	4.32 ± 1.02 a	1127.69 ± 11.51 b

Values are means ± SD, Different letters within the same column indicate significant differences among treatments (*p* < 0.05). SPAD, Soil and Plant Analysis Development chlorophyll meter reading.

**Table 3:** Effects of different treatments on alpha diversity indices of soil bacterial and fungal communities.

Microbes	Treatment	Sobs	Chao1	Ace	Shannon	Simpson
Bacteria	CK	4829.33 ± 158.18 a	5974.41 ± 195.15 a	6086.02 ± 189.05 a	7.176 ± 0.077 a	0.997 ± 0.001 a
	Mi	4373.33 ± 85.01 a	5478.40 ± 96.34 a	5608.77 ± 91.60 a	7.102 ± 0.058 a	0.997 ± 0.000 a
	MM	3624.00 ± 472.49 b	4465.96 ± 572.81 b	4524.29 ± 587.68 b	6.764 ± 0.163 b	0.997 ± 0.000 a
Fungus	CK	340.000 ± 25.239 a	349.62 ± 21.46 a	347.78 ± 23.20 a	4.205 ± 0.270 a	0.964 ± 0.012 a
	Mi	350.333 ± 43.247 a	366.33 ± 46.75 a	362.87 ± 42.72 a	3.501 ± 0.768 a	0.877 ± 0.114 a
	MM	147.333 ± 34.948 b	152.89 ± 32.27 b	152.20 ± 31.47 b	3.390 ± 0.074 a	0.918 ± 0.022 a

Values are means ± SD. Different lowercase letters within the same column and within the same microbial group indicate significant differences among treatments at *p* < 0.05.

## Data Availability

Data is available upon reasonable request.
